# Vaccine hesitancy for COVID19: what is the role of statistical literacy?

**DOI:** 10.3389/fpubh.2023.1230030

**Published:** 2023-09-07

**Authors:** Halle Kahlenberg, Delaney Williams, Miranda A. L. van Tilburg, Michael R. Jiroutek

**Affiliations:** ^1^Department of Pharmaceutical & Clinical Sciences, College of Pharmacy & Health Sciences, Campbell University, Buies Creek, NC, United States; ^2^Jerry M. Wallace School of Osteopathic Medicine, Campbell University, Buies Creek, NC, United States; ^3^Cape Fear Valley Health, Fayetteville, NC, United States; ^4^Marshall University, Huntington, WV, United States

**Keywords:** vaccine hesitancy, statistics, COVID19, statistical literacy, COVID19 vaccine hesitancy

## Abstract

**Introduction:**

Vaccination is an important measure used to control the spread of COVID19. The estimation of risk versus benefit of vaccination is based on the understanding of information about the vaccine. Statistics are frequently part of communications about COVID19. Individuals that do not have an adequate foundation of statistical knowledge may not be able to properly assess associated risks and benefits. This study aims to assess the association between statistical literacy and hesitation to receive the COVID19 vaccine.

**Methods:**

A nationally representative sample of 2,138 adults, recruited through CINT United States, Inc., (Lawrenceville, NJ; http://www.cint.com), completed an internet survey in the summer of 2021. This survey collected demographic measures and information about COVID19 vaccination status. The competency of respondents on various basic statistical concepts was assessed along with the corresponding confidence of respondents in their answers. A multivariable logistic regression model was constructed to assess the relationship between vaccine hesitancy and statistical literacy while controlling for covariates of interest.

**Results:**

Statistical literacy was found to have a negligible association with COVID19 vaccine hesitancy (OR 1.01; 95% CI 1.00–1.02). In addition, differences in the proportion receiving the COVID19 vaccine between political affiliations, income levels, race groups, and ethnicities were observed.

**Discussion:**

The statistical knowledge of the general American public is not commensurate with the need to be literate in basic statistical concepts in the data-driven world in which we live. An effective way to stem vaccine hesitancy may rely on increased statistical knowledge to not be biased by preconceived beliefs shaped by misinformation.

## 1. Introduction

Vaccination is an important measure to control the spread of COVID19. Yet approximately 20% of people are unwilling to be vaccinated (1). The World Health organization Sage Working Group on Vaccine Hesitancy emphasizes that “vaccine hesitancy is complex and is not driven by a simple set of individual factors.” This group emphasizes that factors can be divided into: (1) Confidence or the trust in the political-health system to offer safe and effective vaccines, and (2) complacency, or the perceived risk of the disease (2). These all are related to some form of evaluation of risk perception – the likelihood of getting sick or suffering from complications from the vaccine as well as from COVID19 is identified in multiple studies as one of the main drivers of vaccine hesitancy (3–6). The estimation of risk versus benefit of any vaccination is based on the understanding of information about the vaccine. Statistics are commonly used by media outlets as well as government agencies to communicate the seriousness and spread of COVID19 as well as to convince people to take preventative measures such as mask wearing or being vaccinated. Therefore, statistical literacy is crucial to curb the spread of COVID19.

Education level has been consistently found to be related to vaccine hesitancy (5, 6). For example in a large sample of over 1,800 Americans, the proportion of people unlikely to get the COVID19 vaccine ranged from over 30% in those with less than a high school degree, versus 13% in those with a graduate degree (5) but the specific educational competencies that drive vaccine hesitancy have not been explored. Education, for example, increases critical thinking skills, which inoculates people against mis- or dis-information about vaccines. In addition, those that have received training in statistics may have an advantage in understanding the true risk and benefit of receiving the COVID19 vaccine. Fullone, et al. found that statistical literacy is lower than desired in the general public (7). Even among health sciences faculty, understanding of basic statistical concepts is suspect (8). Statistical literacy has also been found to be lacking among journalists who the public relies on to accurately communicate statistics. According to Malik et al. (9), over 70% of journalism students rated as having poor or fair mathematical skills (9).

Individuals that do not have an adequate foundation of statistical knowledge may not be able to properly assess the risks and benefits of vaccines. This could account for why many people overestimate the risks and underestimate benefits associated with vaccination. Statistical literacy is not an innate skill and must be taught to individuals (10) and thus may be a target for intervention to increase vaccine rates in the population. This study aimed to assess the association between statistical literacy and the hesitation to receive the COVID19 vaccine.

## 2. Materials and methods

### 2.1. Subjects

Participants were recruited throughout the United States using CINT United States, Inc., (Lawrenceville, NJ; http://www.cint.com). CINT has access to a panel of over 3 million participants worldwide. Inclusion criteria were: (1) Resident of the United States, (2) Age 18 or above. The lone exclusion criterion was any individuals ineligible to receive a COVID19 vaccine due to a medical condition or previous allergic reaction per self-report. Quota-based sampling was used to prevent oversampling of the vaccinated population (60% of US at the time of the study). No other restrictions, weighting, etc. were implemented in the sample procedure.

### 2.2. Questionnaires

All participants completed an internet survey in the summer of 2021.

#### 2.2.1. Sample descriptives

The information collected in the survey included demographic and participant characteristics.

#### 2.2.2. Vaccine hesitancy

At the time the survey used in this study was being constructed (spring 2021), vaccine hesitancy related to COVID19 was beginning to appear in the literature. As early as December 2020, Lin et al. (11) had published on the vaccine demand and hesitancy in China (11). As the pandemic progressed, so did the worldwide evaluation of hesitancy. Scoping reviews were done (3, 12), as were broad assessments of hesitancy on public health (13, 14). Subsequent to these broader assessments, investigations of specific populations in regards to hesitancy were undertaken, not surprisingly starting with those in the healthcare field: medical students (4) and healthcare workers (15), followed by populations such as college students (16), youth (17), cancer patients (18), etc. Many of these studies included prospective survey data collection related to hesitancy, but very few were available in the literature in the spring of 2021 and none that we found had any connection to statistical literacy. While there are many ways to assess vaccine hesitancy, which have grown more numerous as the pandemic progressed, the dichotomous simplicity of a straightforward and easily understandable yes/no question seemed to outweigh any limitations. Thus, we elected to use the receipt of the COVID19 vaccination (yes/no by self-report) as a surrogate (marker) for vaccine hesitancy.

#### 2.2.3. Statistical literacy

Sharma (19) provided a thorough literature review of the variety of definitions and models of statistical literacy that have been proposed, finding a wide array of thinking, contexts and terminology on the topic (19). A single, concise and widely agreed upon definition of statistical literacy seems to be a challenge as statistics is so broadly applied across a multitude of fields in the information age in which we live. Given the fact that statistical literacy can have different meanings in different contexts, the definition utilized by Ziegler and Garfield (20) is the one most relevant for the purposes of this study and the one we targeted with our statistical survey questions, specific to COVID19 information found in the news at or near the height of the pandemic (20): “The ability to read, understand, and communicate statistical information. This type of statistical information that is relevant for statistical literacy (e.g., graphical representations, descriptive statistics, inferential statistics) is encountered in daily life, such as in a media article, and involves real contexts.” The term literacy is defined in the Oxford English dictionary as “competence or knowledge in a specified area.” While many of the efforts around statistical literacy focus on determining or assessing a comprehensive understanding of a (not well agreed upon) set of basic statistical ideas/concepts, the term statistical literacy in this study was intended to be implemented along the lines of the Ziegler and Garfield definition as applied to just one specific context – statistical ideas found in the news at the time particular to COVID19. That is, we did not set out to create a tool to assess a breadth of statistical knowledge more widely applicable to any topic at any point in time, but more specifically only to try and assess the knowledge of the general public around statistical ideas in the news at or near the height of the COVID19 vaccination controversy.

With a not-well-agreed-upon definition of statistical literacy in the literature, not surprisingly, a wide variety of disparate measurement tools have been developed. Most broadly, tools such as SfL2011 and PIAAC were developed to assess basic literacy, numeracy and information and communication technology skills (21, 22). More specialized assessments have been created focused on the specific area(s) of interest of the creators. For example, Vicente and Lopez (23) sought to evaluate contextual knowledge official European economic statistics (23). Education and classroom-specific tools such as REALI and BLIS were developed to assess statistical literacy in student populations (24, 25). In fact, in 2017 the Statistics Education Research Journal published an entire special issue on just statistical literacy (26). Another survey tool was developed and utilized in a series of publications to evaluate the basic statistical knowledge of faculty in the health sciences (8, 27–29). However, tools like PARIS21 and SLI were found to be most along the lines of the intent of the current study – designed to assess literacy broadly across a national population, based on archived newspaper articles (30, 31).

In terms of the development of a statistical literacy survey tool for the current study, the timing was the largest challenge. The authors could find no measure of statistical literacy applicable specifically to COVID19. Therefore, in order to attempt to assess statistical knowledge about COVID19-related statistics in the news around the height of the COVID19 pandemic a new assessment needed to be developed. Statements involving basic statistics were extracted from articles about COVID19 in the news at the time (spring 2021) and turned into questions with multiple choice answers. Participants were asked to select the correct answer from among the three provided answers. A total score was calculated by how many questions each participant answered correctly. Face validity was determined by asking multiple faculty researchers from various programs within Campbell University’s College of Pharmacy & Health Sciences to evaluate the accuracy and appropriateness of the questionnaire. Questions (and the multiple choice responses) were revised to improve clarity based on the feedback provided. Some questions were harder than others. For any multiple choice question with three possible choices, a 33% correct response rate would be considered ‘chance’. Only if the correct response rate was higher than 33% would we say that the respondents did better than chance. The percentage correct for three of the 11 questions was at or below chance. In addition, construct validity (how well the questionnaire measured statistical literacy) was checked by asking participants to rate, for each question, how confident they were that their answer to each specific question was correct (Five-point Likert scale: 1 = Extremely unconfident, 2 = Unconfident, 3 = Neutral, 4 = Confident, 5 = Extremely confident). The internal reliability of the confidence questions was strong (standardized Cronbach’s alpha = 0.94 in the current study population). If the questionnaire is valid, the likelihood that a question was answered correctly should be associated with increased confidence. The results in Table 1 support this association. The statistical questions included in the survey can be found in the Appendix.

**Table 1:** Survey statistics questions scoring and confidence.

Question number	Answered correctly *N* (%)	Confidence* N (%)	Number answered correctly out of 11	*N* (%)
–	–	–	0	3 (0.1)
1	1,566 (73.3)	1,576 (73.7)	1	27 (1.3)
2	709 (33.2)	1,128 (52.8)	2	123 (5.8)
3	1,063 (49.7)	1,247 (58.3)	3	240 (11.2)
4	1,444 (67.5)	1,040 (48.6)	4	373 (17.5)
5	684 (32.0)	1,344 (62.9)	5	382 (17.9)
6	974 (45.6)	938 (43.9)	6	362 (16.9)
7	738 (34.5)	834 (39.0)	7	263 (12.3)
8	1,196 (55.6)	1,190 (55.7)	8	206 (9.6)
9	1,095 (51.2)	1,122 (52.5)	9	132 (6.2)
10	428 (20.0)	1,324 (61.9)	10	24 (1.1)
11	1,620 (75.8)	1,430 (66.9)	11	3 (0.1)

*Confidence was defined as those who responded either “Confident” or “Extremely Confident” to the question “How confident are you that your answer to this question is correct?”.

### 2.3. Data analyses

The demographics and survey respondent characteristics were summarized with counts and percentages. Statistical literacy was summarized question by question to report the number and percentage of respondents answering each question correctly as well as cumulatively, reporting the number and percentage of respondents correctly answering 0, 1, 2, …, 11 questions out of the 11 asked. In addition, “Confidence” was defined as those who responded either “Confident” or “Extremely Confident” to each question on the statistical literacy questionnaire and the number and percent reporting ‘Confidence’ were summarized for each statistics question.

Additionally, a multivariable logistic regression model was constructed for the receipt of a COVID19 vaccine to evaluate the predictive value of the percentage score on the 11-question statistical portion of the survey, adjusting for covariates of interest (political affiliation, income level, race group, ethnicity, and age). The interaction term between political affiliation and percentage score was found to be unimportant and dropped from the model. Adjusted odds ratios (ORs) with corresponding 95% Wald confidence intervals (CIs) for each level of each variable included in the model (compared with each variable’s reference group) were reported. The levels utilized for each discrete variable used in the model can be found in Table 2. SAS version 9.4 (SAS Institute Inc., Cary, North Carolina) was used for all analyses.

**Table 2:** Demographics and participant characteristics.

Characteristic	Total *N* (%) *N* = 2,138
Gender Female Male Other	1,099 (51.4) 1,015 (47.5) 24 (1.1)
Race Group Caucasian African American Other Asian Native American or Alaska Native Native Hawaiian or other Pacific Islander	1,147 (67.7) 284 (13.3) 228 (10.7) 141 (6.6) 34 (1.6) 4 (0.2)
Ethnicity Not Hispanic or Latino Hispanic or Latino	1761 (82.4) 377 (17.6)
Education Level Less than high school Some high school, no diploma High school graduate, diploma or equiv. Some college credit, no degree Bachelor’s degree Graduate degree or more	19 (0.9) 68 (3.2) 544 (25.4) 637 (29.8) 565 (26.4) 305 (14.3)
Total Household Income $19,999 or less $20,000 to $49,999 $50,000 to $69,999 $70,000 to $99,999 $100,000 or more Prefer not to say	357 (16.2) 698 (32.7) 369 (17.3) 289 (13.5) 293 (13.7) 132 (6.2)
Political Affiliation Democrat Republican Independent Unaffiliated Prefer not to say	843 (39.4) 530 (24.8) 499 (23.3) 174 (8.1) 92 (4.3)
Number of Statistics Classes Taken 0 Less than one 1 2 3 4+	63 (3.0) 576 (26.9) 502 (23.5) 401 (18.8) 169 (7.9) 427 (20.0)
Received COVID19 Vaccine Yes No	1,449 (67.8) 689 (32.2)

## 3. Results

The study population consisted of 2,138 survey respondents. Sample demographics are given in Table 2 and show about equal number of male/females, predominantly White, non-Hispanic, with the majority of the respondents split across political affiliations. The majority of participants (70%) reported having taken at least one statistics course. Sixty eight percent of the respondents reported receiving the COVID19 vaccine (Table 2).

The percentage answering each statistics question correctly ranged from 20 to 76%, with a mean of 48% over all items (see Table 1). The range of ‘Confidence’ (defined as those who responded either “Confident” or “Extremely Confident” to a question) about answers to the statistics questions ranged from 39 to 73.7% with a mean of 56% over all items (see Table 1). For 8 of the 11 questions the percent with ‘Confidence’ in their answer was higher than the percent answering the corresponding question correctly.

Previously reported factors associated with vaccine hesitancy were tested. All relevant assumptions required to utilize the multivariable logistic regression model constructed were carefully evaluated, with no concerns detected. In addition, the Hosmer and Lemeshow goodness-of-fit test *p*-value was found to be 0.8324 and the model r-square value 0.2198, further suggesting a good model fit to the data and a reasonable accounting of the variability of the outcome due to the predictors included in the model. Receiving the COVID vaccine was associated with being a Democrat vs. Republican (OR = 3.73; 95% CI 2.80–4.99) or Independent (OR = 1.70; 95% CI 1.27–2.27). Wealthier participants were consistently found to have higher odds of receiving the COVID19 than poorer participants across all the income level groups (income level ≥ $100 k vs. $70 k- < $100 k OR = 1.81; 95% CI 1.12–2.92, ≤$20 k vs. $70 k- < $100 k OR = 0.40; 95% CI 0.27–0.60, $20 k- < $50 k vs. $70 k- < $100 k OR = 0.52; 95% CI 0.37–0.75, and $50 k- < $70 k vs. $70 k- < $100 k OR = 0.69; 95% CI 0.46–1.02). The “Other” race group had nearly double the odds of receiving the COVID19 vaccine than the White race group (OR 1.98; 95% CI 1.38–2.85), while the African American race group had 34% lower odds of receiving the COVID19 vaccine than the White race group (OR 0.66; 95% CI 0.47–0.93). Hispanic/Latinos had 56% higher odds of receiving the COVID19 vaccine than non-Hispanic/Latinos (OR 1.56; 95% CI 1.10–2.21), while for each additional year of age, the odds of receiving the COVID19 vaccine increased by 4% (OR 1.04; 95% CI 1.03–1.04).

Given these associations, we tested if receiving the COVID19 vaccine is related to statistical literacy, by using multivariable logistic regression model, while adjusting for political affiliation, income level, race group, ethnicity, and age. Statistical literacy was found to have no meaningful association with COVID19 vaccine hesitancy (OR 1.01; 95% CI 1.00–1.02) (Table 3).

**Table 3:** Multivariable Logistic Regression Model for Receipt of COVID19 Vaccine (Yes/No).

Variables*	Adjusted odds ratio (95% Wald confidence limits)
Stats knowledge score	1.01 (1.0, 1.02)
Political Affiliation: Democrat vs. Republican	3.73 (2.80, 4.99)
Political Affiliation: Independent vs. Republican	1.70 (1.27, 2.27)
Income Level: ≥ $100 k vs. $70 k – < $100 k	1.81 (1.12, 2.92)
Income Level: <$20 k vs. $70 k – < $100 k	0.40 (0.27, 0.60)
Income Level: $20 k – < $50 k vs. $70 k – < $100 k	0.52 (0.37, 0.75)
Income Level: $50 k – < $70 k vs. $70 k – < $100 k	0.69 (0.46, 1.02)
Race: Other† vs. White	1.98 (1.38, 2.85)
Race: African American vs. White	0.66 (0.47, 0.93)
Ethnicity: Hispanic/Latino vs. Non-Hispanic/Latino	1.56 (1.10, 2.21)
Age (years)	1.04 (1.03, 1.04)

Multivariable logistic regression model to predict receipt of COVID vaccine (Yes vs. No).

* Reference group listed last in each row.

† Includes Native American or Alaska Native, Asian, Native Hawaiian or Other Pacific Islander, and “Other”.

## 4. Discussion

The results of this study indicate that the statistical knowledge of the American public is not commensurate with the need to be statistically literate in the data-driven world in which we live. Extrapolating the results of this nationally representative study sample suggest half of Americans would “fail” a multiple-choice assessment of their literacy regarding basic statistical concepts they would be expected to come across in their daily lives, despite a high level of confidence in their knowledge. Logic would dictate that a population which believes they understand what they are reading, but actually does not, may lead to misinformed and incorrect choices being made unknowingly. Confidently proceeding in error has the potential for more devious effects than being unsure if one knows the best path forward and proceeding cautiously while seeking to gain more knowledge on how best to proceed over time. Regardless of the self-perceived confidence level, the low scores on the survey statistics questions intimates a need to address educational shortcomings in statistical literacy.

No meaningful association between statistics knowledge score and willingness to receive the COVID19 vaccine was observed (each percentage point increase in statistics knowledge score predicted, on average, between a zero and 2 % increase in the odds of receiving the COVID19 vaccine, after adjusting for the other factors included in the model). Generally speaking, more statistical knowledge would lead to better assessment of risks and benefits and would be expected to lead to higher likelihood of vaccination. The lack of a stronger predictive effect of increased statistical knowledge reducing people’s hesitancy to receive the COVID vaccine, may show that the evaluation of risk and benefit is not made by cold hard facts and numbers. Rather, factors such as misinformation (and/or disinformation), inaccurate reporting, political allegiances and socio-economic status may be more important in predicting willingness to get vaccinated. This suggests that educational opportunities to increase statistical literacy may have only a small benefit in reducing vaccine hesitancy. Instead, education focused on critical thinking to be able to identify and deal with misinformation may be more beneficial.

This study further suggests discernable differences in willingness to receive the COVID19 vaccine between political affiliations, income levels, racial/ethnic groups and ages. Political ideology, or perhaps just party affiliation appears to influence one’s willingness to receive the COVID19 vaccine, with Democrats estimated to be 273% more likely than Republicans and Independents 81% more likely than Republicans. Democratic political affiliation may be an indicator of positive attitudes toward vaccination, as corroborated by other literature (10).

The model constructed in this study further suggests that as wealth increases, the odds of receipt of the COVID19 vaccine increase. Known disparities in access to and quality of both healthcare and education across income levels may confound the relationship between income level and receipt of the COVID19 vaccine. These disparities can lead to deficits in health literacy, as identified by the deficit in statistical knowledge and health literacy that have been associated with reluctance to receive the COVID19 vaccine in other studies (28, 32).

Disparities in vaccine hesitancy were found to disproportionately affect certain racial and ethnic groups. While Hispanic/Latinos (compared to non-Hispanic/Latinos) and those in the ‘Other’ race group (compared to those in the White race group) had higher odds of receiving the COVID19 vaccine, those in the African American race group had lower odds of receiving the COVID19 vaccine (compared to those in the White race group). Similar results have been previously published, attributed to mistrust of the healthcare system and low health literacy (28, 29). Targeting these racial and ethnic groups to improve health literacy may not only lead to a more favorable attitude toward the COVID19 vaccination, but the knowledge gained may improve the inherent mistrust of the healthcare system which has been identified.

Age appears to have a moderate effect on the odds of receiving the COVID19 vaccine. For each one year increase in age, on average, it is estimated that there exists between a three and four percent increase in the odds of receiving the COVID19 vaccine, after adjusting for the other factors included in the model. As COVID is known to more adversely affect those who are more infirm and infirmities are positively correlated with age, this model result is encouraging for protecting those most potentially adversely effected by this virus (33).

The study has several strengths including a large sample size and results that can be roughly generalized to the American population. In addition, it tests a novel concept, statistical literacy, as a factor in COVID19 vaccination rate. However, it also has several limitations. Given the retrospective nature of the study, no claims can be made for cause and effect. Although, it would be hard to imagine that vaccine hesitancy makes people somehow less statistical literate. Next, we used a newly developed questionnaire for statistical literacy. We provided initial promising face validity, internal consistency, and construct validity. In addition, based on our limited definition of statistical literacy we focused mostly on computational abilities of respondents. If a wider definition of statistical literacy is adopted, a questionnaire should also include other aspects of statistical literacy not assessed in the current study such as interpretation and evaluation of the data. Future studies should examine if these aspects of statistical literacy are more important for vaccine hesitancy. The questionnaire is in need of further testing and development. We also chose whether or not individuals received the COVID19 vaccine as a surrogate of or marker for vaccine hesitancy. The authors acknowledge that receipt of the vaccine may reflect certain circumstances rather than an absence of hesitancy. Similarly, failure to receive the vaccine may not be due solely to hesitancy, but include factors like access, etc. However, the authors felt the benefits of using such a simple and straightforward measure outweighed any limitations. Our findings that receipt of the COVID19 vaccine is associated with several demographic factors, similarly to what is described in the literature on vaccine hesitancy, is encouraging. Lastly, the study was conducted in summer 2021 when the vaccine was new (most people did not get access until spring 2021), and many people were still dying of COVID19 (34, 35). This may have affected the response to our study questions. The study is in need of repetition.

The poor scoring in the statistics questions in the study survey provides ample evidence that significant improvements are needed in statistical literacy of the US public to be able to adequately and accurately read and understand all the information and data we are exposed to daily in this Information Age in which we live. Although no evidence of a clinically meaningful association between statistical literacy and COVID19 vaccine hesitancy was identified, it may be masked by a false sense of overconfidence in the literacy of statistical concepts. In addition, many people may simply ignore newly presented statistical information because of preconceived misaligned perceptions of risk and rewards due to misinformation. In previous studies, both misinformation and political affiliation were associated with COVID19 vaccination rates (33, 36). We found a similar result in our current study with those identifying as Republican (politicians more likely to adhere to and support COVID19 conspiracy beliefs) being less likely to be vaccinated (37). Thus, the way to stem vaccine hesitancy may not be through an increase in statistical knowledge, but rather through battling preconceived beliefs shaped by misinformation.

## Data availability statement

The datasets presented in this article are not readily available because they remain in a secure location, not publicly available. Requests to access the datasets should be directed to MJ, jiroutekm@campbell.edu.

## Ethics statement

The studies involving humans were approved by Campbell University Institutional Review Board. The studies were conducted in accordance with the local legislation and institutional requirements. The participants provided their written informed consent to participate in this study.

## Author contributions

MT and MJ were responsible for the conception and design of the study, data analysis/interpretation, drafting of the article, and approved the final manuscript. HK was responsible for data collection, critical revision of the article, and approved the final manuscript. DW was responsible for data collection and approved the final manuscript. All authors contributed to the article and approved the submitted version.

## Funding

This project was provided by the Campbell University College of Pharmacy & Health Sciences Internal Research Grant program, with funds awarded May 21, 2021.

## Conflict of interest

The authors declare that the research was conducted in the absence of any commercial or financial relationships that could be construed as a potential conflict of interest.

## Publisher’s note

All claims expressed in this article are solely those of the authors and do not necessarily represent those of their affiliated organizations, or those of the publisher, the editors and the reviewers. Any product that may be evaluated in this article, or claim that may be made by its manufacturer, is not guaranteed or endorsed by the publisher.
