# Supplementary material for: Vaccine hesitancy for COVID19: what is the role of statistical literacy?
Source: Front Public Health. 2023 Sep 7;11:1230030. doi: 10.3389/fpubh.2023.1230030 (PMC10513463; doi:10.3389/fpubh.2023.1230030)
Supplement: Supplementary file 1 [file Table_1.docx]

# Appendix

Assessment of Statistics Knowledge Questions (correct answers for each question in bold)

1. “…South Africa expects to get vaccines to inoculate…10% of its population of 60,000,000, from the international COVAX initiative starting in April.” From this information, which of the following the number of South Africans that can expect to get vaccinated?
   1. 600,000,000
   2. 60,000,000
   3. **6,000,000**
2. “The country….that has reported by far the most cases of the coronavirus in Africa, with more than 1.1 million confirmed infections…representing more than 30% of all cases on the continent of 1.3 billion.” From this information, which of the following is the closest estimated number of infections on the continent?
   1. **3,666,667**
   2. 366,667
   3. 3,300,000
3. “On Monday, the rate of positive tests was 33.3% — the highest recorded since the outbreak began. Health officials want to get that number below 5%.”. The ‘rate of positive tests of 33.3%...’ can be best described to mean which of the following?
   1. One out of three people (33.3%) received a test to determine if they were positive.
   2. **One out of three (33.3%) of all tests given in a specified period of time was positive.**
   3. One out of three people (33.3%) tested positive.
4. “In Los Angeles County, which saw its case numbers increase by 905 percent since Nov. 1, 10 people are getting sick with Covid-19 every minute…”. A 905 percent increase in case numbers can be best described to mean which of the following?
   1. **Case numbers have increased by more than 10-fold (i.e., more than a factor of 10).**
   2. Case numbers have increased by 905 cases.
   3. Case numbers have nearly doubled.
5. “We have a highly efficacious vaccine, 94% to 95% effective. A 94% to 95% effective vaccine can best be described to mean which of the following?
   1. 94% to 95% of the people vaccinated will not get the illness.
   2. **The percentage of those who get the illness is reduced by 94 to 95%.**
   3. 5% to 6% of the people will get the illness.
6. “There are currently 361,148 deaths confirmed to be caused by Covid-19 in the US. With an estimated population of 322,000,000, that equals to...” a rate of death of about
   1. 361 deaths out of 100,000 Americans.
   2. **112 deaths out of 100,000 Americans.**
   3. 322 deaths out of 100,000 Americans.
7. “With more than 21 million cases and 361,123 deaths in the US… The global coronavirus case total is 87m, with 1.9m people dying in just over a year since the start of the pandemic.” Based on the provided numbers, the US accounts for approximately
   1. one in ten fatalities worldwide
   2. one in four fatalities worldwide
   3. **one in five fatalities worldwide.**
8. “The seven-day average for new cases per day is 2,715...” A seven-day average for new cases can best be described as which of the following?
   1. **The sum of number of new cases that have occurred on each of the previous seven days, divided by 7.**
   2. The number of new cases that have occurred on each of the previous seven days.
   3. The number of new cases that have occurred on any one day in the previous week times seven.
9. “The seven-day average for new cases per day increased from 2,715 to 2,996 on Saturday.” The seven-day average in this example increasing from 2,715 new cases to 2,996 new cases can best be described as which of the following?
   1. **The average number of new cases for each previous seven-day period is generally increasing.**
   2. The number of new cases is increasing each and every day for the previous seven days.
   3. 281 new cases were observed in the last seven days.
10. “The coronavirus pandemic has taken an extraordinarily heavy toll on New York State, where … 1 in every 500 residents has now died of Covid-19”. Which of the following is the best definition of one in every 500 residents having died of Covid-19?
    1. On average, 1 out of every 500 residents has died of Covid-19.
    2. At least 500 people need to be tested to observe a single death.
    3. **Amongst every sample or collection of 500 residents, one has died of Covid-19.**
11. "Blood group O is significantly associated with reduced susceptibility to SARS-CoV-2 infection," the study authors wrote….” The significant association with reduced susceptibility to SARS-CoV-2 reported here can be most accurately interpreted to mean that
    1. people with blood type O are not less likely to be infected with SARS-CoV-2.
    2. **people with type O blood are less likely to become infected with SARS-CoV-2.**
    3. people with blood type O will not become infected with SARS-CoV-2 (are immune).
